# Evaluating the structure of commensalistic epiphyte–phorophyte networks: a comparative perspective of biotic interactions

**DOI:** 10.1093/aobpla/plz011

**Published:** 2019-03-07

**Authors:** Carlos Naranjo, José M Iriondo, María L Riofrio, Carlos Lara-Romero

**Affiliations:** 1 Departamento de Ciencias Biológicas, Universidad Técnica Particular de Loja (UTPL), Loja, Ecuador; 2 Biodiversity and Conservation Area, School of Experimental Sciences (ESCET), Rey Juan Carlos University (URJC), Madrid, Spain; 3 Global Change Research Group, Mediterranean Institute of Advanced Studies (CSIC–IUB), Mallorca, Balearic Islands, Spain

**Keywords:** Ecological interactions, modularity, nestedness, orchids, specialization, tropics

## Abstract

Epiphytic vascular plants comprise an essential part of the tropical flora and are a key component for ecosystem functioning. Some recent studies have used a network approach to investigate the interaction of epiphytes with host phorophytes at the community level. However, knowledge on commensalistic epiphyte–phorophyte network structure still lags behind with regard to other biotic interaction networks. Our goal was to provide a more complete overall perspective on commensalistic epiphyte–phorophyte interaction and its placement with respect to other better studied mutualistic interactions. We hypothesized that the intensity of the fitness effect of the different types of biotic interactions would determine the degree of specialization of the interacting organisms. Thus, commensalistic epiphyte–phorophyte interactions would have lower specialization than mutualistic interactions. We compiled and analysed the structural properties (nestedness, network specialization and modularity) of 12 commensalistic epiphyte–phorophyte networks and compared them with the same metrics to 11 ant–myrmecophyte, 86 pollination and 13 seed dispersal mutualistic networks. Epiphyte–phorophyte networks were nested and modular with regard to the corresponding null models and had greater nestedness than mutualistic networks, whereas specialization and modularity were significantly lower. Commensalistic epiphyte–phorophyte networks of interactions are both nested and modular, and hence, are structured in a similar way to most other types of networks that involve co-evolutionary interactions. Nevertheless, the nature and intensity of the ecological processes involved in the generation of these patterns is likely to differ. The lower values of modularity in commensalistic epiphyte–phorophyte networks are probably due to the low levels of specialization and the lack of co-evolutionary processes between the interacting partners.

## Teaser

The goal of the study is to provide an overall perspective on epiphyte–phorophyte networks and their placement with respect to the networks of other more studied biotic interactions. The results showed that epiphyte–phorophyte networks are structured similarly to most other types of networks that involve co-evolutionary interactions as they were, in most cases, significantly nested and modular. It also confirmed that the interaction between vascular epiphytes and host phorophytes is predominantly generalist.

## Introduction

The establishment of biotic interactions is a topic of great interest in ecology. For a long time, ecologists have searched for patterns and processes related to species distribution and interspecific interactions in communities (Janzen 1974; Thompson 1999; Wisz *et al.* 2013). These interactions can be described as networks in which each species is connected to one or more different species (Bascompte *et al.* 2003; Bascompte and Jordano 2014). The network structure has relevant implications for the coexistence and stability of species and has been described as the architecture of biodiversity (Bascompte and Jordano 2007). The topological properties of these networks provide information on community organization and offer a general framework to evaluate the different types of interactions between species (Proulx *et al.* 2005; Blick and Burns 2009; Bascompte and Jordano 2014). Furthermore, network analysis has been widely used to measure ecological specialization (Blüthgen *et al.* 2007; Schleuning *et al.* 2012; Lara-Romero *et al.* 2016; Carstensen *et al.* 2018; Dugger *et al.* 2019; but see Mello *et al.* 2015). Several types of mutualistic (Bascompte *et al.* 2003; Bascompte and Jordano 2007; Bascompte 2009; Vázquez *et al.* 2009; Fortuna *et al.* 2010; Pastor and Garca 2015) and antagonistic networks (Cagnolo *et al.* 2011; Hagen *et al.* 2012; Elias *et al.* 2013; Morris *et al.* 2014) have been extensively studied contributing to an understanding of the factors influencing the structure of these networks.

Two widely accepted topological properties of these networks, with theoretical and practical implications, are nestedness (Bascompte *et al.* 2003; Thébault and Fontaine 2010) and modularity (Olesen *et al.* 2007; Thébault and Fontaine 2010). The first implies that specialist species interact with a proper subset of the partners that interact with generalist species (Bascompte *et al.* 2003), whereas the second denotes a pattern in which some subsets (modules) of species are more linked to each other than to species in other modules (Fortuna *et al.* 2010). Nestedness and modularity are linked to community stability and robustness against perturbations and environmental changes (Thébault and Fontaine 2010; Dalsgaard *et al.* 2013; Robinson *et al.* 2015; Nuwagaba *et al.* 2017). However, how these properties emerge and what is their relevance to network stability, is still under debate, especially for nestedness (Burgos *et al.* 2007; Minoarivelo and Hui 2016; Landi *et al.* 2018). Although nestedness and modularity have traditionally been considered antagonistic properties of networks (Fortuna *et al.* 2010), some studies emerged in the past years have shown that they are not mutually exclusive, and that they can interact with one another (Flores *et al.* 2013; Pinheiro *et al.* 2016).

Interactions between vascular epiphytes (henceforth referred to as epiphytes) and host trees (phorophytes) are considered to be commensalistic because epiphytes establish on the host tree for support without causing harm or benefit (Zotz 2016). Epiphytic vascular plants comprise an essential part of the tropical and subtropical flora (Kreft *et al.* 2004; Krömer *et al.* 2005) and are a key component for ecosystem functioning and diversity (Kreft *et al.* 2004). Despite their importance, our understanding of the mechanisms structuring epiphyte communities is still rather poor (Burns and Zotz 2010; Wagner *et al.* 2015). This gap is in part due to the lack of a rigorous theoretical and empirical framework that could guide investigations of epiphyte assemblages (Burns and Zotz 2010; Wagner *et al.* 2015). Yet in recent years, novel studies have used a network approach to investigate these interactions at the community level. They have found that the composition of epiphyte–phorophyte interactions appears to be structured deterministically, as happens with mutualistic and antagonistic interactions. Specifically, they have found that these networks are highly nested (Blick and Burns 2009; Silva *et al.* 2010; Sáyago *et al.* 2013; Ceballos *et al.* 2016; but see Burns and Zotz 2010), even more than in mutualistic and antagonistic networks (Piazzon *et al.* 2011), and that there is no phylogenetic signal in species interaction patterns (Silva *et al.* 2010). These studies also support that the host specificity of epiphytes is small and that most interactions occur among generalist epiphytes and generalist phorophytes (Laube and Zotz 2006; Silva *et al.* 2010; Sáyago *et al.* 2013). However, varying traits in host phorophytes, such as bark texture and size and temporal and spatial distribution of species, can have significant effects on the performance of epiphyte species (Hietz 1999; Zotz *et al.* 1999; Callaway *et al.* 2002; Laube and Zotz 2006), and therefore, they have been invoked to determine the degree of epiphyte host specificity (Wagner *et al.* 2015). This is particularly important because there is a long-standing and ongoing discussion on whether vascular epiphytes show host specificity (Cornelissen and Steege 1989; Benzing 1990; Laube and Zotz 2006; Vergara-Torres *et al.* 2010, reviewed in Wagner *et al.* 2015).

Despite progress made in recent years, the knowledge on the structure of commensalistic epiphyte–phorophyte networks lags behind those of other biotic interaction networks (Sáyago *et al.* 2013) and thorough comparisons between the former and latter are missing. This is in part because network studies in these commensalistic interactions are still scarce and scattered and both data source and the methodology differ across studies, making it difficult to detect general patterns (but see Piazzon *et al.* 2011). Furthermore, modularity has not been yet characterized for this type of interaction, and therefore, we do not know the specific importance of this basic network build-up mechanism. To provide a more complete overall perspective on epiphyte–phorophyte networks and their placement with respect to the networks of other more studied mutualistic interactions, we analysed 12 epiphyte–phorophyte networks, and compared them with the same metrics to ant–myrmecophyte (plant–ant), pollination and seed dispersal mutualistic networks. Our general goal is to understand how epiphyte–phorophyte networks are structured. Mutualistic networks were selected as a baseline for comparison because their overall degree of specialization covers a broad range (Blüthgen *et al.* 2007). Pollination mutualisms tend to be more specialized than seed dispersal mutualisms, probably due to evolutionary considerations (Wheelwright and Orians 1982; Blüthgen *et al.* 2007), whereas obligate-myrmecophytic symbioses represent one of the most specialized mutualistic interactions (Blüthgen *et al.* 2007). Characterizing ecological specialization is challenging, because it entails different definitions and can be assessed at different scales (Blüthgen 2010; Devictor *et al.* 2010; Poisot *et al.* 2011). Here we considered specialization as a niche-breath correlate, which can be estimated for species interacting at the community level by well-established network metrics (Blüthgen *et al.* 2006; Dormann 2011; Poisot *et al.* 2012). Considering that the different types of biotic interactions have distinctive effects on the fitness of the interacting organisms, we hypothesized that the magnitude of fitness change generated by the different types of biotic interactions would determine the degree of specialization of the interacting organisms. Thus, organisms involved in biotic interactions which greatly affect their fitness would have greater specialization. Since epiphytes are assumed to establish on their hosts without impairing their fitness (Zotz 2016), we predicted that the epiphyte–phorophyte interaction would entail low degree of specialization. Moreover, considering that specialization and host specificity is negatively associated with nestedness (Bascompte *et al.* 2003) and positively associated with modularity (Albrecht *et al.* 2014; Dormann and Strauss 2014), we also predicted that epiphyte–phorophyte networks would be more generalist and nested, but less modular than the mutualistic ones. To the best of our knowledge, this is the first study to compare the modularity of epiphyte–host tree networks with that of mutualistic networks. Our study greatly expands the scope of Piazzon *et al.* (2011) who carried out a comparative study about nestedness that only included epiphyte–phorophyte networks of the same study region.

## Materials and Methods

### Data set

To compare epiphyte–phorophyte networks with mutualistic networks, we compiled a data set of 122 interaction networks **[see****Supporting Information—Table S1****]**. We restricted data to tropical and subtropical areas to minimize potential biases in topological metrics due to heterogeneous environmental conditions. Twelve published data sets that measured quantitatively interactions between vascular epiphyte and host species in tropical and subtropical areas were included in the study (Table 1). Epiphyte species vary across studies, but they are distributed mainly among the families Orchidaceae and Bromeliaceae **[see****Supporting Information—Table S1****]**. The data set also included 86 pollination, 13 seed dispersal and 11 ant–myrmecophyte (plant–ant) networks from published studies considering only studies carried out in tropical and subtropical areas, available in Web of life (http://www.web-of-life.es) and additional works **[see****Supporting Information—Table S1****]**. The main characteristics of the analysed networks are described in **Supporting Information—Table S1**; detailed description of data sets and field sampling procedures can be found in the attached references.

**Table 1.** Network properties of 12 epiphyte–phorophyte networks analysed in this study. TMF, tropical montane forest; TDF, tropical dry forest; LIF, low inundated forest; TRF, temperate rainforest; ECF, evergreen cloud forest; NODF, nestedness index; *H*′2, specialization index; *Q*, modularity; GR, ground-based survey; CA, canopy-based survey. Network size is the sum of epiphytes and phorophytes. ^a^Denotes that data were obtained from the original source. *na* denotes that data were not available from the original source and bipartite matrix was not published and therefore we could not estimate the metric. Values that are statistically significant from random expectations (*Z*-test: *P* < 0.05) are indicated in bold.

Reference	Locality	Habitat	Field sampling	Lat	Long	Network size	NODF	*H*′2	*Q*
Ceballos *et al.* (2016)	Tucumán, Argentina	TMF	GR	−26.76	−65.33	44	**69.53**	0.11	**0.18**
Dejean *et al.* (1995)	Quintana Roo, Mexico	LIF	*na*	19.38	−87.79	20	**67.77**	0.24	**0.22**
Laube and Zotz (2006)	San Lorenzo, Panamá	TDF	CA, GR	8.3	−82.1	107	**20.81**	0.28	**0.28**
Martínez-Meléndez *et al.* (2008)	Cerro Quetzal, Mexico	ECF	CA	15.72	−92.92	25	59.89	0.10	**0.13**
C. Naranjo, unpubl. data	Zamora, Ecuador	TMF	CA	−3.99	−76.1	146	**56.98**	0.21	**0.17**
Piazzon *et al.* (2011)	Caulin Forest, Chile	TRF	GR	−41.83	−73.6	17	64.29	0.09	0.11
Piazzon *et al.* (2011)	Senda Darwin, Chile	TRF	GR	−41.88	−73.67	16	**74.65**	0.09	0.09
Piazzon *et al.* (2011)	Llanquihue, Chile	TRF	GR	−41.85	−73.57	22	**74.54**	0.06	0.08
Piazzon *et al.* (2011)	Quilar, Chile	TRF	GR	−41.92	−73.60	19	68.95	0.12	**0.12**
Sáyago *et al.* (2013)	Jalisco, Mexico	TDF	GR	20.66	−103.5	62	**62.89** ^**a**^	0.23^a^	*na*
Vergara-Torres *et al.* (2010)	San Andrés Cal, Mexico	TDF	CA, GR	18.95	−99.08	16	**76.67**	0.1	**0.09**
Zhao *et al.* (2015)	Xishuangbanna, China	TMF	GR	22.01	100.8	180	**16.4** ^**a**^	0.5^a^	*na*

### Data analysis

For each of the 122 networks, data were completely reanalysed to compute the following network metrics: (i) nestedness, (ii) complementary specialization and (iii) modularity. Nestedness quantifies the degree to which species with few interactions are connected to highly connected species (Bascompte *et al.* 2003). Nestedness was evaluated with the nestedness metric based on overlap and decreasing fill (NODF) index (Almeida-Neto *et al.* 2008). As the NODF metric is dependent on network size and sampling intensity (Ulrich *et al.* 2009), the significance of NODF was evaluated against a fixed-fixed null distribution derived from 1000 random networks with the same number of nodes and interactions as the observed networks. To characterize, network specialization at the community level, we used the index of complementary specialization *H′*2, which quantifies the degree of niche divergence of elements within an entire bipartite network, that is, whether species in a network tend to partition or share their interaction partners (Blüthgen *et al.* 2006). It ranges from 0 (low specialization, high niche overlap) to 1 (high specialization, low niche overlap). Modularity detects the degree to which the network is structured as cohesive subgroups of species (modules) in which the density of interactions is higher within subgroups than among subgroups (Dormann and Strauss 2014). Modularity was estimated using the *QuaBiMo* algorithm (*Q*), which is based on a hierarchical random graph approach, adapted for quantitative bipartite networks (Dormann and Strauss 2014). As the algorithm is a stochastic process, results may vary among computations. For each network, we therefore ran the *QuaBiMo* algorithm 10 times and retained the optimal modular configuration, i.e. the iteration with highest *Q* value. Because *Q* also can be affected by network size (Dormann and Strauss 2014), the significance of *Q* was assessed against a null distribution derived from 100 random networks generated as for NODF. The value of modularity ranges between 0 (random network with no modules) and 1 (maximum modularity). Modularity increases with increasing link density within modules and decreasing connectedness between different modules (i.e. as specialization increases) (Albrecht *et al.* 2014; Dormann and Strauss 2014). Thus, we expected considerable lower *Q* values in epiphyte–phorophyte networks compared to mutualistic networks due to higher generalization. NODF calculations were based on the binary data matrix and *H′*2 and *Q* were all computed based on interaction frequencies (quantitative matrix). NODF calculations were based on the binary data matrix because weighted metrics explicitly assume that nested patterns are determined by the abundance of interacting species (Almeida-Neto and Ulrich 2011), and thereby it is not in accordance with the classical concept of nestedness, more focused on assessing the role of species as specialist and generalist. In any case, we also estimated weighted NODF, finding that NODF and wNODF were highly correlated (Pearson’s correlation *r* = 0.86, *P* < 0.0001, *n* = 118) and yielded similar results in comparisons across types of biotic interactions **[see****Supporting Information—Fig. S1****]**.

To characterize specialization at the species level, we quantified the roles of species within networks with two species-level metrics: complementary specialization (*d′*) and between-module connector values (*c*-values). The two metrics define a specialist as an organism having stronger link strengths in association with a limited subset of its possible resources (Poisot *et al.* 2012). The metric *d′* measures the level of specialization of each species based on its discrimination from a random selection of partners (Blüthgen *et al.* 2006). It is analogous to the calculation of *H*′2 at the community level and ranges from 0 (no specialization, species that interact with their partners proportionally to their availability) to 1 (perfect specialists, species that disproportionately interact with rare partners) (Blüthgen *et al.* 2006). *C*-value determines the importance of a species in connecting different modules by interactions with species from other modules, thereby reducing modularity (e.g. Schleuning *et al.* 2014; Dugger *et al.* 2019). It has a 0 value if all interactions of a species are within its own module and it is close to 1 if the interactions of a species are evenly distributed among modules. Network analyses were conducted in R v. 3.1.2 (R Development Core Team 2011) with the add-on libraries bipartite v. 2.04 (Dormann *et al.* 2008) and vegan v. 2.4-5 package (Oksanen *et al.* 2007). The network structure was explored and visualized with the R package igraph (Csardi and Nepusz 2006) using the Fruchterman–Reingold force-directed layout algorithm (Fruchterman and Reingold 1991), which is an energy-minimization plotting algorithm that optimizes the placement of the species in the graph.

To contrast network- and species-level metrics among epiphyte–phorophyte, pollination, seed dispersal and ant–myrmecophyte networks, we applied linear mixed models (LMMs). All models included metrics as the response variables, type of interaction as a fixed factor and locality as random factor to control for potential spatial non-independence (Zuur *et al.* 2009). Network size was included as a continuous predictor variable in models fitted for network-level metrics, given that network metrics can depend on network size or sampling intensity (Blüthgen *et al.* 2008; Ulrich *et al.* 2009; Dormann and Strauss 2014). We assumed Gaussian error for all LMMs, and NODF was ln-transformed to reach normality and homoscedasticity. Model residuals were checked graphically for normality and homogeneity of variances using diagnostic plots (Zuur *et al.* 2009). Using the *Anova* function in the *R* package ‘car’, we tested the null hypothesis that the response means are identical across type of interaction. When the overall analyses of variance indicated a significant difference (*P* < 0.05), a Tukey’s test for *post hoc* multiple contrasts was conducted using glht in the package multcomp v. 1.4-6 (Hothorn *et al.* 2008). Models were assessed for goodness-of-fit to the data using the marginal (*Rm*^2^) and conditional (*Rc*^2^) *R*^2^ described by Nakagawa and Schielzeth (2013). *Rm*^2^ represents the variance explained by fixed factors while *Rc*^2^ is interpreted as variance explained by both fixed and random factors (i.e. the entire model). Linear mixed models were fitted with the R package ‘lme4’ (Bates *et al.* 2015).

## Results

**Figure 1.**
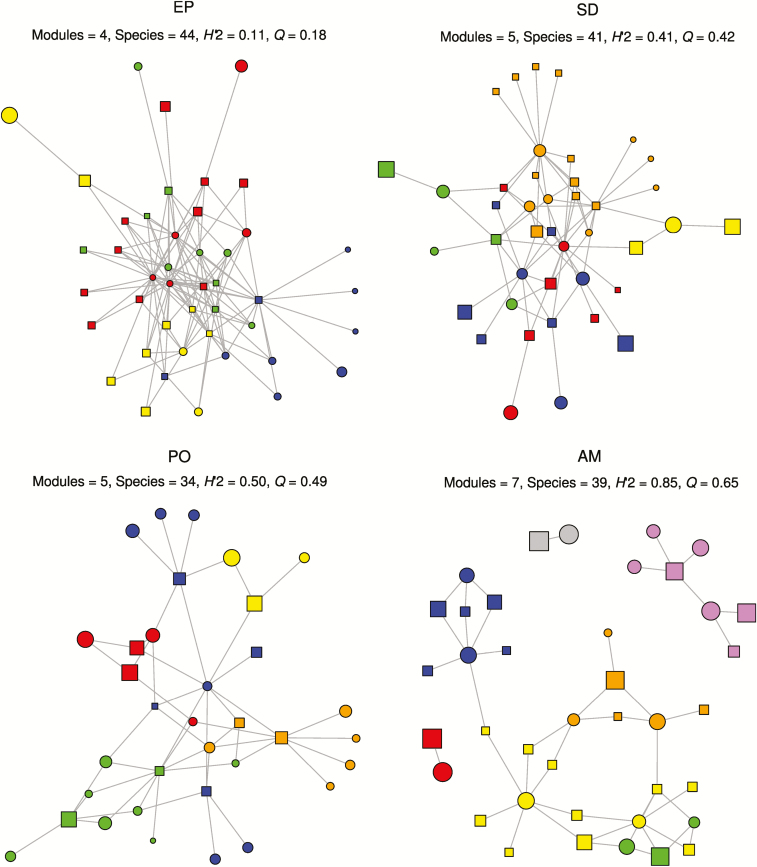
Visualization of representative networks of similar size for each type of biotic interaction shows marked differences of modularity and specialization. Node shape distinguishes the two interacting groups involved in each network. In the epiphyte–phorophyte network, squares and circles denote epiphytes and host trees, respectively. In the mutualistic networks, squares denote animals and circles represent plants. The grey lines linking the two levels represent pairwise species interactions. The colour of each node indicates the module to which the species belongs. Node size represents complementary specialization (*d′*) of each species. EP, epiphyte–phorophyte network (Ceballos *et al.* 2016); SD, seed dispersal network (Carlo *et al.* 2003); PO, pollination network (Kaiser-Bunbury *et al.* 2014); AM, ant–myrmecophyte network (Fonseca and Ganade 1996).

**Figure 2.**
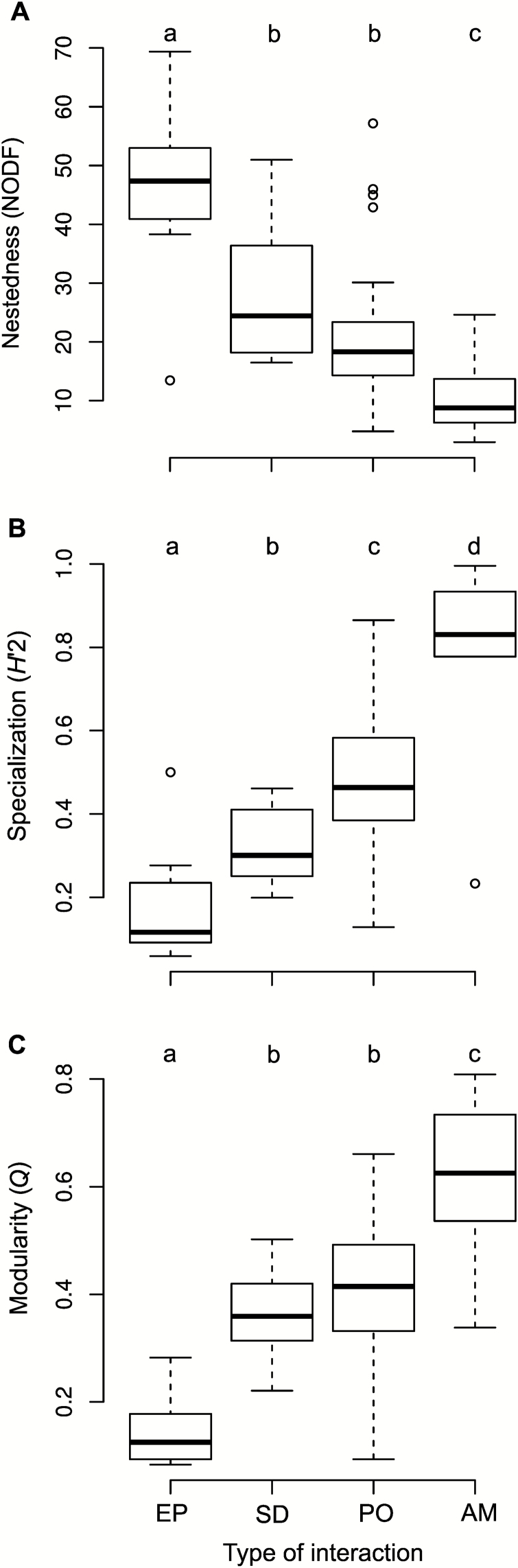
Variation in network metrics across network interaction types: commensalistic epiphyte–phorophyte (EP), seed dispersal (SD), pollination (PO) and ant–myrmecophyte (AM) networks. (A) Nestedness; (B) specialization; (C) modularity. Different letters denote significant differences among network types (*P*-value < 0.05) after Tukey’s correction for multiple comparisons.

**Figure 3.**
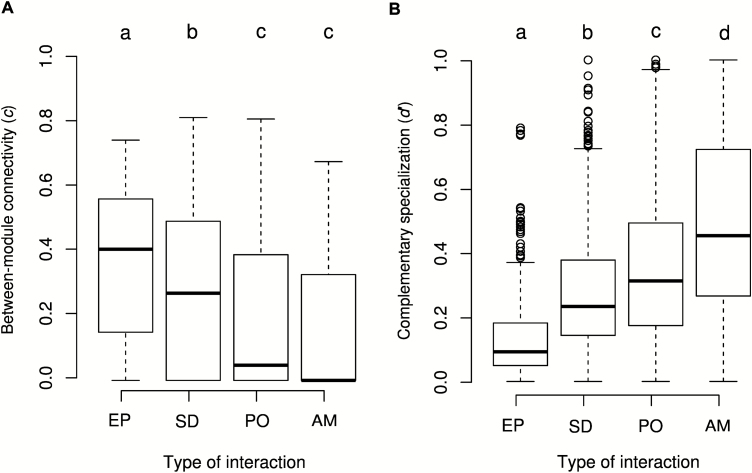
Variation of species niche-based metrics across network interaction types: commensalistic epiphyte–phorophyte (EP), seed dispersal (SD), pollination (PO) and ant–myrmecophyte (AM) networks. (A) Between-module connectivity; (B) complementary specialization. Different letters denote significant differences among network types (*P*-value < 0.05) after Tukey’s correction for multiple comparisons.

All three network metrics varied significantly among the different types of interactions (all LMM tests; *P* < 0.0001; Figs 1 and 2; **see****Supporting Information—Table S2**). Specialization and modularity were significantly lower in the epiphyte–phorophyte networks than in the rest of the networks (Figs 1 and 2, all tests: *P* < 0.016), whereas nestedness was significantly higher (Fig. 2, *P* < 0.02). Network size influenced NODF values (LMM tests: *P* < 0.01; **see****Supporting Information—Table S2**), but did not significantly affect *H′*2 and *Q* (LMM tests: all *P* > 0.13; **see****Supporting Information—Table S2**). *Rc*^2^, which represents variance explained by the entire model, ranged between 0.59 and 0.62 **[see****Supporting Information—Table S2****]**, with the variance explained by fixed factors (*Rm*^2^) representing a large fraction in all cases (41–67 %). Regarding species-level metrics, *d′* and *c*-values varied significantly among types of interactions (Fig. 3, *d′*: χ^2^ = 74.93, *df* = 3, *P* < 0.0001, *Rc*^2^ = 0.19, *Rm*^2^ = 0.1; *c*-value: χ^2^ = 100.5, *df* = 3, *P* < 0.0001, *Rc*^2^ = 0.26, *Rm*^2^ = 0.13). *C*-value was significantly higher in the epiphyte–phorophyte networks than in the rest of the networks (Fig. 3, all tests: *P* < 0.0001), whereas *d’* was significantly lower (Fig. 3, all tests *P* < 0.0001).

Focusing on epiphyte–phorophyte networks, the three network metrics varied considerably (NODF = 44.44 ± 18.05, *H′*2 = 0.18 ± 0.13, *Q* = 0.15 ± 0.06; Table 1). NODF values were significantly higher than those from the null models in 9 of 12 analysed networks, while *Q* was significantly higher than random expectations in 7 of the 10 analysed networks (Table 1).

## Discussion

In this paper, we evaluated the network structure of 12 epiphyte–phorophyte networks and compared it to those found in ant–myrmecophyte (plant–ant), pollination and seed dispersal mutualistic networks. Results confirmed our hypothesis that epiphyte–phorophyte networks are more nested and connected and have lower modularity and specialization than mutualistic networks.

Most studies agree that nestedness observed in epiphyte–phorophyte networks is largely explained by differences in species abundance, i.e. species interact randomly resulting in interaction frequencies that are proportional to relative species abundances (Burns 2007; Silva *et al.* 2010; Piazzon *et al.* 2011; Sáyago *et al.* 2013; Ceballos *et al.* 2016). This is further supported by the low values of the index of network specialization (*H′*2) found in our study, which suggests that colonization of phorophytes by epiphytes is proportional to the abundance of phorophytes (Blüthgen *et al.* 2007). However, 9 out of 12 networks analysed were more nested than null model expectations, which implies that nestedness is not entirely explained by abundance. This is in line with previous case studies where reported nested patterns were attributed to ecological processes (Burns 2007; Silva *et al.* 2010; Piazzon *et al.* 2011; Sáyago *et al.* 2013; Ceballos *et al.* 2016). In any case, current observational data are rather controversial, and there is still no general agreement about the key processes shaping nestedness in epiphyte–phorophyte interactions, extending the debate observed in other types of interaction networks (Burgos *et al.* 2007; Minoarivelo and Hui 2016; Pinheiro *et al.* 2016).

Modularity (*Q*) differed from null expectations, which indicated a certain level of ecological compartmentalization. Epiphyte networks therefore appear to be structured similarly to most other types of species interactions as they showed both significant nestedness and modularity (Bascompte *et al.* 2003; Olesen *et al.* 2007; Thébault and Fontaine 2010). Modularity implies that some subsets (modules) of species are more linked to each other than to species in other modules. This is the first time that modularity is reported for epiphyte–phorophyte networks, and so far, we lack information about the mechanisms that underpin modularity. In mutualistic interactions, modules have been usually viewed as potential co-evolutionary units of biological significance that arise due to trophic specialization, divergent selection regimes and phylogenetic clustering of closely related species (Olesen *et al.* 2007; Martín-González *et al.* 2012; Watts *et al.* 2016; Morente-López *et al.* 2018). These mechanisms are not plausible in epiphyte–phorophyte networks because interactions among species are not mutually advantageous and therefore, natural selection does not favour the convergence and complementarity of traits in interacting species. This is further supported by the lack of evidence of phylogeny as an explanatory factor structuring epiphyte–phorophyte networks (Silva *et al.* 2010; Sáyago *et al.* 2013). However, although there is not strong specialization, varying traits in host phorophytes do provide contrasting microhabitat conditions that influence the interactions with epiphytes (Hietz 1999; Zotz *et al.* 1999; Callaway *et al.* 2002; Laube and Zotz 2006). Differential use of these resources can give rise to phorophyte-specific epiphyte spectra (Zotz *et al.* 1999). We propose that modularity may arise as a result of ‘structural host specificity’ (*sensu*Wagner *et al.* 2015), i.e. differences in the performance of the focal epiphyte species on a given host phorophyte relative to another host phorophyte. Hence, modules may contain a set of species with convergent traits, which influence epiphyte performance. These traits may be related to physical bark characteristics, leaf and bark chemistry or branch architecture of the phorophytes and microclimatic specificity, duration of life cycle, size at maturity or diaspore characteristics of the epiphytes (Wagner *et al.* 2015). Identification of modules may bring us closer to an understanding of the structure of complex networks of interaction (Olesen *et al.* 2007). Thus, a more explicit focus on modules as study objects may open pathways towards a better mechanistic understanding of the epiphyte–phorophyte interaction.

Our results provide insight on the current discussion of whether vascular epiphytes show low levels of specialization (Wagner *et al.* 2015). Species-level metrics indicated lower degree of specialization in epiphyte–phorophyte than in mutualistic networks, consistent with our results at the network level. Nested structure indicates that host specificity of vascular epiphytes is small and instead, specialists engaged in few interactions are connected predominantly with generalists (Silva *et al.* 2010; Piazzon *et al.* 2011; Taylor *et al.* 2016). This implies that strict specialization is rare. Furthermore, low values found for *H′*2 and *Q* in epiphyte–phorophyte networks compared to mutualistic networks indicated high niche overlap among species or modules (Blüthgen *et al.* 2006; Schleuning *et al.* 2014; Dugger *et al.* 2019). This is not surprising because driving forces for co-evolution are expected to be weaker in epiphyte–phorophyte interaction as epiphytes do not have fitness effects on their host. Furthermore, epiphytes are structurally dependent plants that cannot actively search for appropriate phorophytes and thus only have the possibility of establishing at the location where diaspores are deposited by chance (Wagner *et al.* 2015). This leads to concluding that a general pattern of host generality is the rule (Wagner *et al.* 2015).

There are some possible sources of variation that cannot be tested in our study due to the limited number of networks available. For example, we were not able to assess differences in network structure across types of habitat within tropical regions. Studies examining vascular epiphyte communities are particularly scarce outside neotropic and most of the studies were performed in tropical dry forest (Burns and Zotz 2010; Silva *et al.* 2010; Sáyago *et al.* 2013). This is important because, for example, specialization of vascular epiphytes may differ between dry forests and rainforests (Vergara-Torres *et al.* 2010) altering the interaction structure. However, the robustness of our results, along with the similar outcomes found in other regions (Burns 2008), reinforces the idea that commensalistic epiphyte–phorophyte networks are deterministically structured. Another factor to consider is the effect of sampling strategies, i.e. ground- vs. canopy-based inventories, on network structure. Both sampling methods provide different effectiveness in recording species richness and frequency (Flores-Palacios and García-Franco 2001). Again, the limited number of studies available prevent us from making any formal analysis. Previous comparisons between ground-based and canopy-based inventories did not identify substantial effects on network structure (Burns 2008). Yet, sampling bias cannot be ruled-out entirely and comparisons between ground-based and canopy-based inventories should be carried out to identify potential biases that might result from the use of different methods. Finally, several of the networks analysed were comprised of only one type of epiphyte (i.e. orchids, bromeliads or angiosperm epiphytes). This may lead to a phylogenetic bias in the network metric values because different types of epiphytes can use different strategies to establish, grow and reproduce (Zotz and Schmidt 2006), which can affect the interaction structure. This may be particularly important when only certain phylogenetic groups of a richer epiphyte–phorophyte community are considered, which may entail important consequences for measurement of network structure (Lewinsohn *et al.* 2006; Flores *et al.* 2013).

## Conclusions

This study showed that commensalistic epiphyte–phorophyte networks are more nested, but less modular, than the mutualistic ones. Overall, these findings confirmed that the interaction between vascular epiphytes and host phorophytes is predominantly generalist. The study also identified for first time that epiphyte–phorophyte networks of interactions are both nested and modular, and hence, are structured in a similar way to most other types of networks that involve co-evolutionary interactions. A better understanding of underpinning mechanisms that drive nestedness and modularity is required to gain insight on the epiphyte–phorophyte interaction. Further research is also needed to evaluate the generalization of our results across regions and systems, and to identify the factors responsible for the convergence between epiphyte–phorophyte and mutualistic networks.

## Data

The data sets generated during and/or analysed during the current study are available in the Figshare Digital Repository: https://doi.org/10.6084/m9.figshare.7751189.v1. The R-code can be downloaded from GitHub at https://github.com/CarlosLaraR/R-ecology.

## Sources of Funding

This research was partially supported by Senescyt – Secretaría de Educación Superior, Ciencia, Tecnología e Innovación (Ecuador). C.L.-R. was supported by a Juan de la Cierva-Formación postdoctoral fellowship (FJCI-2015-24712).

## Contributions by the Authors

The work was designed by C.N., M.L.R. and C.L.-R.; C.L.-R. analysed the results with the contribution of C.N. and J.M.I.; C.L.-R., J.M.I. and C.N. wrote the manuscript with the contribution of M.L.R.

## Conflict of Interest

None declared.

## Supporting Information

The following additional information is available in the online version of this article—

**Table S1.** Bibliographic reference, taxonomic focus, location and topological properties of the epiphyte–phorophyte and mutualistic networks analysed in this study.

**Table S2.** Linear mixed models performed to contrast metrics among type of interactions.

**Figure S1.** Distribution of weighted NODF values across network interaction types.

Supplementary Materials

## References

[CIT0001] Albrecht M, Padrón B, Bartomeus I, Traveset A 2014 Consequences of plant invasions on compartmentalization and species’ roles in plant-pollinator networks. Proceedings of the Royal Society B: Biological Sciences 281:20140773. 10.1098/rspb.2014.0773 PMC4083793 24943368

[CIT0002] Almeida-Neto M, Guimarães P, Guimarães PR, Loyola RD, Ulrich W 2008 A consistent metric for nestedness analysis in ecological systems: reconciling concept and measurement. Oikos 117:1227–1239.

[CIT0003] Almeida-Neto M, Ulrich W 2011 A straightforward computational approach for measuring nestedness using quantitative matrices. Environmental Modelling & Software 26:173–178.

[CIT0004] Bascompte J 2009 Mutualistic networks. Frontiers in Ecology and the Environment 7:429–436.

[CIT0005] Bascompte J, Jordano P 2007 Plant-animal mutualistic networks: the architecture of biodiversity. Annual Review Ecology Evolution and Systematic 38:567–593.

[CIT0006] Bascompte J, Jordano P 2014 Mutualistic networks, 1st edn. Princeton, NJ: Princeton University Press.

[CIT0007] Bascompte J, Jordano P, Melian CJ, Olesen JM 2003 The nested assembly of plant-animal mutualistic networks. Proceedings of National Academy of Science 100:9383–9387. 10.1073/pnas.1633576100 PMC170927 12881488

[CIT0008] Bates D, Mächler M, Bolker B, Walker S 2015 Fitting linear mixed-effects models using lme4. Journal of Statistical Software 67:1–48.

[CIT0009] Benzing D 1990 Vascular epiphytes: general biology and related biota, 1st edn. Cambridge, UK: Cambridge University Press.

[CIT0010] Blick R, Burns K 2009 Network properties of arboreal plants: are epiphytes, mistletoes and lianas structured similarly? Perspectives in Plant Ecology, Evolution and Systematics 11:41–52.

[CIT0011] Blüthgen N 2010 Why network analysis is often disconnected from community ecology: a critique and an ecologist’s guide. Basic and Applied Ecology 11:185–195.

[CIT0012] Blüthgen N, Fründ J, Vázquez DP, Menzel F 2008 What do interaction network metrics tell us about specialization and biological traits? Ecology 89:3387–3399. 19137945 10.1890/07-2121.1

[CIT0013] Blüthgen N, Menzel F, Blüthgen N 2006 Measuring specialization in species interaction networks. BMC Ecology 6:9. 16907983 10.1186/1472-6785-6-9 PMC1570337

[CIT0014] Blüthgen N, Menzel F, Hovestadt T, Fiala B, Blüthgen N 2007 Specialization, constraints, and conflicting interests in mutualistic networks. Current Biology 17:341–346. 17275300 10.1016/j.cub.2006.12.039

[CIT0015] Burgos E, Ceva H, Perazzo RP, Devoto M, Medan D, Zimmermann M, María Delbue A 2007 Why nestedness in mutualistic networks? Journal of Theoretical Biology 249:307–313. 17897679 10.1016/j.jtbi.2007.07.030

[CIT0016] Burns KC 2007 Network properties of an epiphyte metacommunity. Journal of Ecology 95:1142–1151.

[CIT0017] Burns KC 2008 Meta-community structure of vascular epiphytes in a temperate rainforest. Botany 86:1252–1259.

[CIT0018] Burns KC, Zotz G 2010 A hierarchical framework for investigating epiphyte assemblages: networks, meta-communities, and scale. Ecology 91:377–385. 20392003 10.1890/08-2004.1

[CIT0019] Cagnolo L, Salvo A, Valladares G 2011 Network topology: patterns and mechanisms in plant-herbivore and host-parasitoid food webs. The Journal of Animal Ecology 80:342–351. 21143226 10.1111/j.1365-2656.2010.01778.x

[CIT0020] Callaway RM, Reinhart KO, Moore GW, Moore DJ, Pennings SC 2002 Epiphyte host preferences and host traits: mechanisms for species-specific interactions. Oecologia 132:221–230. 28547355 10.1007/s00442-002-0943-3

[CIT0021] Carlo TA, Collazo JA, Groom MJ 2003 Avian fruit preferences across a Puerto Rican forested landscape: pattern consistency and implications for seed removal. Oecologia 134:119–131. 12647189 10.1007/s00442-002-1087-1

[CIT0022] Carstensen DW, Trøjelsgaard K, Ollerton J, Morellato LPC 2018 Local and regional specialization in plant–pollinator networks. Oikos 127:531–537.

[CIT0023] Ceballos SJ, Chacoff NP, Malizia A 2016 Interaction network of vascular epiphytes and trees in a subtropical forest. Acta Oecologica 77:152–159.

[CIT0024] Cornelissen JHC, Steege HT 1989 Distribution and ecology of epiphytic bryophytes and lichens in dry evergreen forest of Guyana. Journal of Tropical Ecology 5:131–150.

[CIT0025] Csardi G, Nepusz T 2006 The igraph software package for complex network research. Inter Journal, Complex Systems 1695:1–10.

[CIT0026] Dalsgaard B, Trøjelsgaard K, Martín González A, Nogués-Bravo D, Ollerton J, Petanidou T, Sandel B, Schleuning M, Wang Z, Rahbek C, Sutherland W, Svenning J, Olesen J 2013 Historical climate-change influences modularity and nestedness of pollination networks. Ecography 36:1331–1340.

[CIT0027] Dejean A, Olmsted I, Snelling RR 1995 Tree-epiphyte-ant relationships in the low inundated forest of Sian Ka’an Biosphere Reserve, Quintana Roo, Mexico. Biotropica 27:57–70.

[CIT0028] Devictor V, Clavel J, Julliard R, Lavergne S, Mouillot D, Thuiller W, Venail P, Villéger S, Mouquet N 2010 Defining and measuring ecological specialization. Journal of Applied Ecology 47:15–25.

[CIT0029] Dormann CF 2011 How to be a specialist? Quantifying specialisation in pollination networks. Network Biology 1:1–20.

[CIT0030] Dormann CF, Gruber B, Fründ J 2008 Introducing the bipartite package: analyzing ecological networks. R News 8:8–11.

[CIT0031] Dormann CF, Strauss R 2014 A method for detecting modules in quantitative bipartite networks. Methods in Ecology and Evolution 5:90–98.

[CIT0032] Dugger PJ, Blendinger PG, Böhning-Gaese K, Chama L, Correia M, Dehling M, Emer C, Farwing N, Fricke E, Galetti M, García D, Grass I, Heleno R, Jacomassa F, Moraes S, Moran C, Muñoz M, Neuschulz E, Nowak L, Piratelli A, Pizo M, Quitián M, Rogers H, Ruggera R, Saavedra F, Sánchez M, Sánchez R, Santillán V, Schabo D, Ribeiro da Silva F, Timóteo S, Traveset A, Vollstädt M, Schleuning M 2019 Seed-dispersal networks are more specialized in the Neotropics than in the Afrotropics. Global Ecology and Biogeography 28:248–261.

[CIT0033] Elias M, Fontaine C, van Veen FJ 2013 Evolutionary history and ecological processes shape a local multilevel antagonistic network. Current Biology 23:1355–1359. 23791729 10.1016/j.cub.2013.05.066

[CIT0034] Flores CO, Valverde S, Weitz JS 2013 Multi-scale structure and geographic drivers of cross-infection within marine bacteria and phages. The Isme Journal 7:520–532. 23178671 10.1038/ismej.2012.135 PMC3578562

[CIT0035] Flores-Palacios A, García-Franco JG 2001 Sampling methods for vascular epiphytes: their effectiveness in recording species richness and frequency. Selbyana 22:181–191.

[CIT0036] Fonseca CR, Ganade G 1996 Asymmetries, compartments and null interactions in an Amazonian ant-plant community. Journal of Animal Ecology 66:339–347.

[CIT0037] Fortuna MA, Stouffer DB, Olesen JM, Jordano P, Mouillot D, Krasnov BR, Poulin R, Bascompte J 2010 Nestedness versus modularity in ecological networks: two sides of the same coin? The Journal of Animal Ecology 79:811–817. 20374411 10.1111/j.1365-2656.2010.01688.x

[CIT0038] Fruchterman TMJ, Reingold EM 1991 Graph drawing by force-directed placement. Software: Practice and Experience 21:1129–1164.

[CIT0039] Hagen M, Kissling WD, Rasmussen C, De Aguilar M, Brown L, Carstensen D, Alves-Dos-Santos I, Dupont Y, Edwards F, Genini J, Guimarães P, Jenkins G, Jordano P, Kaiser-Bunbury C, Ledger M, Maia K, Marquitti F, Mclaughlin Ó, Morellato P, O’Gorman E, Trøjelsgaard K, Tylianakis J, Vidal M, Woodward G, Olesen J 2012 Biodiversity, species interactions and ecological networks in a fragmented world. Advances in Ecological Research 46:89–120.

[CIT0040] Hietz P 1999 Diversity and conservation of epiphytes in a changing environment. Pure and Applied Chemistry 70:2114–2125.

[CIT0041] Hothorn T, Bretz F, Westfall P 2008 Simultaneous inference in general parametric models. Biometrical Journal 50:346–363. 18481363 10.1002/bimj.200810425

[CIT0042] Janzen DH 1974 The deflowering of central America. Natural History of New York 83:48–53.

[CIT0043] Kaiser-Bunbury CN, Vázquez DP, Stang M, Ghazoul J 2014 Determinants of the microstructure of plant-pollinator networks. Ecology 95:3314–3324.

[CIT0044] Kreft H, Köster N, Küper W, Nieder J, Barthlott W 2004 Diversity and biogeography of vascular epiphytes in Western Amazonia, Yasuní, Ecuador. Journal of Biogeography 31:1463–1476.

[CIT0045] Krömer T, Kessler M, Robbert G, Acebey A 2005 Diversity patterns of vascular epiphytes along an elevational gradient in the Andes. Journal of Biogeography 32:1799–1809.

[CIT0046] Landi P, Minoarivelo HO, Brännström Å, Hui C, Dieckmann U 2018 Complexity and stability of adaptive ecological networks: a survey of the theory in community ecology. In: Mensah P, Katerere D, Hachigonta S, Roodt A, eds. Systems analysis approach for complex global challenges. Cham, Switzerland: Springer International Publishing, 209–248.

[CIT0047] Lara-Romero C, García C, Morente-López J, Iriondo JM 2016 Direct and indirect effects of shrub encroachment on alpine grasslands mediated by plant–flower visitor interactions. Functional Ecology 30:1521–1530.

[CIT0048] Laube S, Zotz G 2006 Neither host-specific nor random: vascular epiphytes on three tree species in a Panamanian lowland forest. Annals of Botany 97:1103–1114. 16574691 10.1093/aob/mcl067 PMC2803392

[CIT0049] Lewinsohn TM, Inácio Prado P, Jordano P, Bascompte J, Olesen JM 2006 Structure in plant–animal interaction assemblages. Oikos 113:174–184.

[CIT0050] Martínez-Meéndez N, Pérez-Farrera MA, Flores-Palacios A 2008 Estratificación vertical y preferencia de hospedero de las epífitas vasculares de un bosque nublado de Chiapas, México. Revista de Biologia Tropical 56:2069–2086. 19419102

[CIT0051] Martín-González AM, Allesina S, Rodrigo A, Bosch J 2012 Drivers of compartmentalization in a Mediterranean pollination network. Oikos 121:2001–2013.

[CIT0052] Mello MAR, Rodrigues FA, Costa L, Kissling W, Şekercioğlu Ç, Marquitti F, Kalko E 2015 Keystone species in seed dispersal networks are mainly determined by dietary specialization. Oikos 124:1031–1039.

[CIT0053] Minoarivelo HO, Hui C 2016 Trait-mediated interaction leads to structural emergence in mutualistic networks. Evolutionary Ecology 30:105–121.

[CIT0054] Morente-López J, Lara-Romero C, Ornosa C, Iriondo JM 2018 Phenology drives species interactions and modularity in a plant - flower visitor network. Scientific Reports 8:9386. 29925965 10.1038/s41598-018-27725-2 PMC6010405

[CIT0055] Morris RJ, Gripenberg S, Lewis OT, Roslin T 2014 Antagonistic interaction networks are structured independently of latitude and host guild. Ecology Letters 17:340–349. 24354432 10.1111/ele.12235 PMC4262010

[CIT0056] Nakagawa S, Schielzeth H 2013 A general and simple method for obtaining R2 from generalized linear mixed-effects models. Methods in Ecology and Evolution 4:133–142.

[CIT0057] Nuwagaba S, Zhang F, Hui C 2017 Robustness of rigid and adaptive networks to species loss. PLoS One 12:e0189086. 29216245 10.1371/journal.pone.0189086 PMC5720727

[CIT0058] Oksanen J, Kindt R, Legendre P, O’Hara B, Simpson G, Solymos P, Stevens M, Wagner H 2007 The vegan package. Community Ecology Package 10:631–637.

[CIT0059] Olesen JM, Bascompte J, Dupont YL, Jordano P 2007 The modularity of pollination networks. Proceedings of National Academy of Science 104:19891–19896. 10.1073/pnas.0706375104 PMC2148393 18056808

[CIT0060] Pastor JM, Garca FJ 2015 Dragging in mutualistic networks. Networks and Heterogeneous Media 10:37–52.

[CIT0061] Piazzon M, Larrinaga AR, Santamaría L 2011 Are nested networks more robust to disturbance? A test using epiphyte-tree, comensalistic networks. PLoS One 6:e19637. 21589931 10.1371/journal.pone.0019637 PMC3092765

[CIT0062] Pinheiro RB, Félix GM, Chaves AV, Lacorte GA, Santos FR, Braga ÉM, Mello MA 2016 Trade-offs and resource breadth processes as drivers of performance and specificity in a host-parasite system: a new integrative hypothesis. International Journal for Parasitology 46:115–121. 26552015 10.1016/j.ijpara.2015.10.002

[CIT0063] Poisot T, Bever JD, Nemri A, Thrall PH, Hochberg ME 2011 A conceptual framework for the evolution of ecological specialisation. Ecology Letters 14:841–851. 21699641 10.1111/j.1461-0248.2011.01645.x PMC3152695

[CIT0064] Poisot T, Canard E, Mouquet N, Hochberg ME 2012 A comparative study of ecological specialization estimators. Methods in Ecology and Evolution 3:537–544.

[CIT0065] Proulx SR, Promislow DE, Phillips PC 2005 Network thinking in ecology and evolution. Trends in Ecology & Evolution 20:345–353. 16701391 10.1016/j.tree.2005.04.004

[CIT0065a] R Development Core Team. 2011 R: A language and environment for statistical computing. R Foundation for Statistical Computing, Vienna http://www.R-project.org.

[CIT0066] Robinson KM, Hauzy C, Loeuille N, Albrectsen BR 2015 Relative impacts of environmental variation and evolutionary history on the nestedness and modularity of tree-herbivore networks. Ecology and Evolution 5:2898–2915. 26306175 10.1002/ece3.1559 PMC4541994

[CIT0067] Sáyago R, Lopezaraiza-Mikel M, Quesada M, Álvarez-Añorve MY, Cascante-Marín A, Bastida JM 2013 Evaluating factors that predict the structure of a commensalistic epiphyte–phorophyte network. Proceedings of the Royal Society B: Biological Sciences 280:20122821. 10.1098/rspb.2012.2821 PMC3574374 23407832

[CIT0068] Schleuning M, Fründ J, Klein AM, Abrahamczyk S, Alarcón R, Albrecht M, Andersson GK, Bazarian S, Böhning-Gaese K, Bommarco R, Dalsgaard B, Dehling DM, Gotlieb A, Hagen M, Hickler T, Holzschuh A, Kaiser-Bunbury CN, Kreft H, Morris RJ, Sandel B, Sutherland WJ, Svenning JC, Tscharntke T, Watts S, Weiner CN, Werner M, Williams NM, Winqvist C, Dormann CF, Blüthgen N 2012 Specialization of mutualistic interaction networks decreases toward tropical latitudes. Current Biology 22:1925–1931. 22981771 10.1016/j.cub.2012.08.015

[CIT0069] Schleuning M, Ingmann L, Strauss R, Fritz SA, Dalsgaard B, Matthias Dehling D, Plein M, Saavedra F, Sandel B, Svenning JC, Böhning-Gaese K, Dormann CF 2014 Ecological, historical and evolutionary determinants of modularity in weighted seed-dispersal networks. Ecology Letters 17:454–463. 24467289 10.1111/ele.12245

[CIT0070] Silva IA, Ferreira AG, Lima M, Soares J 2010 Networks of epiphytic orchids and host trees in Brazilian gallery forests. Journal of Tropical Ecology 26:127–137.

[CIT0071] Taylor A, Saldaña A, Zotz G, Kirby C, Díaz I, Burns K 2016 Composition patterns and network structure of epiphyte–host interactions in Chilean and New Zealand temperate forests. New Zealand Journal of Botany 54:204–222.

[CIT0072] Thébault E, Fontaine C 2010 Stability of ecological communities and the architecture of mutualistic and trophic networks. Science 329:853–856. 20705861 10.1126/science.1188321

[CIT0073] Thompson JN 1999 The evolution of species interactions. Science 284:2116–2118. 10381869 10.1126/science.284.5423.2116

[CIT0074] Ulrich W, Almeida-Neto M, Gotelli NJ 2009 A consumer’s guide to nestedness analysis. Oikos 118:3–17.

[CIT0075] Vázquez DP, Chacoff NP, Cagnolo L 2009 Evaluating multiple determinants of the structure of plant-animal mutualistic networks. Ecology 90:2039–2046. 19739366 10.1890/08-1837.1

[CIT0076] Vergara-Torres CA, Pacheco-Álvarez MC, Flores-Palacios A 2010 Host preference and host limitation of vascular epiphytes in a tropical dry forest of central Mexico. Journal of Tropical Ecology 26:563–570.

[CIT0077] Wagner K, Mendieta-Leiva G, Zotz G 2015 Host specificity in vascular epiphytes: a review of methodology, empirical evidence and potential mechanisms. AoB Plants 7:plu092; doi:10.1093/aobpla/plu092. 25564514 PMC4306756

[CIT0078] Watts S, Dormann CF, Martín González AM, Ollerton J 2016 The influence of floral traits on specialization and modularity of plant-pollinator networks in a biodiversity hotspot in the Peruvian Andes. Annals of Botany 118:415–429. 27562649 10.1093/aob/mcw114 PMC4998976

[CIT0079] Wheelwright NT, Orians GH 1982 Seed dispersal by animals: contrasts with pollen dispersal, problems of terminology, and constraints on coevolution. The American Naturalist 119:402–413.

[CIT0080] Wisz MS, Pottier J, Kissling WD, Pellissier L, Lenoir J, Damgaard CF, Dormann CF, Forchhammer MC, Grytnes JA, Guisan A, Heikkinen RK, Høye TT, Kühn I, Luoto M, Maiorano L, Nilsson MC, Normand S, Öckinger E, Schmidt NM, Termansen M, Timmermann A, Wardle DA, Aastrup P, Svenning JC 2013 The role of biotic interactions in shaping distributions and realised assemblages of species: implications for species distribution modelling. Biological Reviews of the Cambridge Philosophical Society 88:15–30. 22686347 10.1111/j.1469-185X.2012.00235.x PMC3561684

[CIT0081] Zhao M, Geekiyanage N, Xu J, Khin MM, Nurdiana DR, Paudel E, Harrison RD 2015 Structure of the epiphyte community in a tropical montane forest in SW China. PLoS One 10:e0122210. 25856457 10.1371/journal.pone.0122210 PMC4391920

[CIT0082] Zotz G 2016 Plants on plants-the biology of vascular epiphytes, 1st edn. Cham, Switzerland: Springer International.

[CIT0083] Zotz G, Bermejo P, Dietz H 1999 The epiphyte vegetation of *Annona glabra* on Barro Colorado Island, Panama. Journal of Biogeography 26:761–776.

[CIT0084] Zotz G, Schmidt G 2006 Population decline in the epiphytic orchid *Aspasia principissa*. Biological Conservation 129:82–90.

[CIT0085] Zuur A, Ieno E, Walker N, Saveliev A, Smith G 2009 Mixed effects models and extensions in ecology with R, 1st edn. New York: Springer-Verlag.

