# Supplementary material for: Evaluating the structure of commensalistic epiphyte–phorophyte networks: a comparative perspective of biotic interactions
Source: AoB Plants. 2019 Mar 7;11(2):plz011. doi: 10.1093/aobpla/plz011 (PMC6462211; doi:10.1093/aobpla/plz011)
Supplement: Supplementary Materials [file plz011_suppl_supplementary_materials.pdf]

Table S1. Bibliographic reference, taxonomic focus, location and topological properties of the epiphyte-phorophyte and mutualistic networks analysed in this study. NODF, nestedness index; H'2, specialization index; Q, modularity. Network size is the sum of epiphytes and phorophytes. [a] denotes that data was obtained from the original source. *na* denotes that data was not available from the original source and therefore we could not estimate the metric.

Number	Reference	Taxonomic focus	Locality	Lat	Long	Network size	NODF	H´2	Q
Pollination									
1	Abreu and Vieira [1]	(several families)	Southeastern Brazil	-20.7500	-42.9200	22	35.49	0.43	0.39
2	Bezerra et al.[2]	Bee-Malpighiaceae	Northeastern Brazil	-8.5050	-37.2014	26	19.93	0.20	0.22
3	Canela[3]	(several families)	Southeastern Brazil	-22.5000	-44.8300	36	27.35	0.55	0.40
4	del Coro and Ornelas[4]	(several families)	Mexico	19.5000	-105.0500	26	84.93	0.57	0.44
5	Gutierrez and Rojas[5]	(several families)	Southeastern Colombia	1.2500	-77.4300	40	33.48	0.48	0.44
6	Ingversen[6]	(several families)	Jamaica	18.3539	-77.6453	72	42.13	0.60	0.63
7	Ingversen[6]	(several families)	Syndicate, Dominica	15.5189	-61.4672	74	36.67	0.46	0.48
8	Kaiser-Bunbury et al.[7]	(several families)	Mauritius	-20.7000	57.7333	50	26.53	0.41	0.44
9	Kaiser-Bunbury et al.[7]	(several families)	Mauritius	-20.7000	57.7333	50	17.30	0.36	0.37
10	Kaiser-Bunbury et al.[7]	(several families)	Mauritius	-20.7000	57.7333	58	14.56	0.38	0.39
11	Kaiser-Bunbury et al.[7]	(several families)	Mauritius	-20.7000	57.7333	67	23.40	0.43	0.44
12	Kaiser-Bunbury et al.[7]	(several families)	Mauritius	-20.7000	57.7333	87	24.79	0.50	0.57
13	Kaiser-Bunbury et al.[7]	(several families)	Mauritius	-20.7000	57.7333	71	19.11	0.58	0.60
14	Kaiser-Bunbury et al.[7]	(several families)	Mauritius	-20.7000	57.7333	68	24.47	0.33	0.48
15	Kaiser-Bunbury et al.[7]	(several families)	Mauritius	-20.7000	57.7333	47	26.90	0.59	0.61
16	Kaiser-Bunbury et al.[7]	(several families)	Mauritius	-20.7000	57.7333	58	28.94	0.61	0.59

17	Kaiser-Bunbury et al.[7]	(several families)	Mauritius	-20.7000	57.7333	39	33.74	0.61	0.54
18	Kaiser-Bunbury et al.[7]	(several families)	Mauritius	-20.7000	57.7333	34	39.70	0.56	0.57
19	Kaiser-Bunbury et al.[7]	(several families)	Mauritius	-20.7000	57.7333	37	41.56	0.45	0.50
20	Kaiser-Bunbury et al.[7]	(several families)	Mauritius	-20.7000	57.7333	38	32.11	0.64	0.51
21	Kaiser-Bunbury et al.[7]	(several families)	Mauritius	-20.7000	57.7333	48	30.12	0.41	0.40
22	Kaiser-Bunbury et al.[7]	(several families)	Mauritius	-20.7000	57.7333	51	31.79	0.41	0.39
23	Kaiser-Bunbury et al.[7]	(several families)	Mauritius	-20.7000	57.7333	56	39.79	0.41	0.44
24	Kaiser-Bunbury et al.[7]	(several families)	Mauritius	-20.7000	57.7333	52	31.96	0.46	0.49
25	Kaiser-Bunbury et al.[7]	(several families)	Mauritius	-20.7000	57.7333	48	30.06	0.49	0.48
26	Kaiser-Bunbury et al.[7]	(several families)	Mauritius	-20.7000	57.7333	31	33.71	0.51	0.48
27	Kaiser-Bunbury et al.[7]	(several families)	Mauritius	-20.7000	57.7333	30	33.18	0.50	0.56
28	Kaiser-Bunbury et al.[7]	(several families)	Mauritius	-20.7000	57.7333	28	18.30	0.54	0.53
29	Kaiser-Bunbury et al.[7]	(several families)	Mauritius	-20.7000	57.7333	44	29.28	0.47	0.49
30	Kaiser-Bunbury et al.[7]	(several families)	Mauritius	-20.7000	57.7333	39	46.46	0.51	0.56
31	Kaiser-Bunbury et al.[7]	(several families)	Mauritius	-20.7000	57.7333	38	33.25	0.64	0.66
32	Kaiser-Bunbury et al.[8]	(several families)	Mahé, Seychelles	-4.6667	55.4333	17	36.76	0.64	0.46
33	Kaiser-Bunbury et al.[8]	(several families)	Mahé, Seychelles	-4.6667	55.4333	22	35.39	0.32	0.32
34	Kaiser-Bunbury et al.[8]	(several families)	Mahé, Seychelles	-4.6667	55.4333	20	30.90	0.35	0.27
35	Kaiser-Bunbury et al.[8]	(several families)	Mahé, Seychelles	-4.6667	55.4333	22	45.89	0.27	0.30
36	Kaiser-Bunbury et al.[8]	(several families)	Mahé, Seychelles	-4.6667	55.4333	34	42.35	0.37	0.35
37	Kaiser-Bunbury et al.[8]	(several families)	Mahé, Seychelles	-4.6667	55.4333	35	19.31	0.32	0.32
38	Kaiser-Bunbury et al.[8]	(several families)	Mahé, Seychelles	-4.6667	55.4333	32	27.38	0.25	0.23

39	Kaiser-Bunbury et al.[8]	(several families)	Mahé, Seychelles	-4.6667	55.4333	25	25.62	0.42	0.36
40	Kaiser-Bunbury et al.[8]	(several families)	Mahé, Seychelles	-4.6667	55.4333	16	41.57	0.69	0.56
41	Kaiser-Bunbury et al.[8]	(several families)	Mahé, Seychelles	-4.6667	55.4333	19	47.79	0.58	0.49
42	Kaiser-Bunbury et al.[8]	(several families)	Mahé, Seychelles	-4.6667	55.4333	18	25.00	0.65	0.38
43	Kaiser-Bunbury et al.[8]	(several families)	Mahé, Seychelles	-4.6667	55.4333	19	36.29	0.28	0.26
44	Kaiser-Bunbury et al.[8]	(several families)	Mahé, Seychelles	-4.6667	55.4333	26	42.92	0.37	0.40
45	Kaiser-Bunbury et al.[8]	(several families)	Mahé, Seychelles	-4.6667	55.4333	17	15.50	0.56	0.40
46	Kaiser-Bunbury et al.[8]	(several families)	Mahé, Seychelles	-4.6667	55.4333	24	24.68	0.30	0.23
47	Kaiser-Bunbury et al.[8]	(several families)	Mahé, Seychelles	-4.6667	55.4333	27	28.68	0.30	0.25
48	Kaiser-Bunbury et al.[8]	(several families)	Mahé, Seychelles	-4.6667	55.4333	22	18.29	0.68	0.58
49	Kaiser-Bunbury et al.[8]	(several families)	Mahé, Seychelles	-4.6667	55.4333	29	28.77	0.59	0.57
50	Kaiser-Bunbury et al.[8]	(several families)	Mahé, Seychelles	-4.6667	55.4333	32	17.76	0.47	0.45
51	Kaiser-Bunbury et al.[8]	(several families)	Mahé, Seychelles	-4.6667	55.4333	25	24.06	0.71	0.64
52	Kaiser-Bunbury et al.[8]	(several families)	Mahé, Seychelles	-4.6667	55.4333	29	40.28	0.30	0.28
53	Kaiser-Bunbury et al.[8]	(several families)	Mahé, Seychelles	-4.6667	55.4333	28	46.82	0.73	0.56
54	Kaiser-Bunbury et al.[8]	(several families)	Mahé, Seychelles	-4.6667	55.4333	36	38.69	0.46	0.40
55	Kaiser-Bunbury et al.[8]	(several families)	Mahé, Seychelles	-4.6667	55.4333	29	21.31	0.39	0.33
56	Kaiser-Bunbury et al.[8]	(several families)	Mahé, Seychelles	-4.6667	55.4333	16	38.94	0.44	0.39
57	Kaiser-Bunbury et al.[8]	(several families)	Mahé, Seychelles	-4.6667	55.4333	20	36.63	0.52	0.42
58	Kaiser-Bunbury et al.[8]	(several families)	Mahé, Seychelles	-4.6667	55.4333	19	47.51	0.65	0.36
59	Kaiser-Bunbury et al.[8]	(several families)	Mahé, Seychelles	-4.6667	55.4333	27	42.38	0.41	0.39
60	Kaiser-Bunbury et al.[8]	(several families)	Mahé, Seychelles	-4.6667	55.4333	26	27.20	0.46	0.42

61	Kaiser-Bunbury et al.[8]	(several families)	Mahé, Seychelles	-4.6667	55.4333	20	29.17	0.46	0.43
62	Kaiser-Bunbury et al.[8]	(several families)	Mahé, Seychelles	-4.6667	55.4333	24	17.91	0.34	0.33
63	Kaiser-Bunbury et al.[8]	(several families)	Mahé, Seychelles	-4.6667	55.4333	24	31.43	0.36	0.28
64	Kaiser-Bunbury et al.[8]	(several families)	Mahé, Seychelles	-4.6667	55.4333	10	31.87	0.57	0.21
65	Kaiser-Bunbury et al.[8]	(several families)	Mahé, Seychelles	-4.6667	55.4333	22	31.48	0.73	0.55
66	Kaiser-Bunbury et al.[8]	(several families)	Mahé, Seychelles	-4.6667	55.4333	24	35.58	0.34	0.30
67	Kaiser-Bunbury et al.[8]	(several families)	Mahé, Seychelles	-4.6667	55.4333	30	36.94	0.44	0.38
68	Kaiser-Bunbury et al.[8]	(several families)	Mahé, Seychelles	-4.6667	55.4333	33	18.65	0.43	0.42
69	Kaiser-Bunbury et al.[8]	(several families)	Mahé, Seychelles	-4.6667	55.4333	30	28.57	0.45	0.38
70	Kaiser-Bunbury et al.[8]	(several families)	Mahé, Seychelles	-4.6667	55.4333	35	18.06	0.33	0.31
71	Kaiser-Bunbury et al.[8]	(several families)	Mahé, Seychelles	-4.6667	55.4333	13	42.50	0.78	0.46
72	Kaiser-Bunbury et al.[8]	(several families)	Mahé, Seychelles	-4.6667	55.4333	17	28.52	0.54	0.33
73	Kaiser-Bunbury et al.[8]	(several families)	Mahé, Seychelles	-4.6667	55.4333	25	22.79	0.51	0.42
74	Kaiser-Bunbury et al.[8]	(several families)	Mahé, Seychelles	-4.6667	55.4333	21	38.56	0.42	0.31
75	Kaiser-Bunbury et al.[8]	(several families)	Mahé, Seychelles	-4.6667	55.4333	34	28.98	0.50	0.49
76	Kaiser-Bunbury et al.[8]	(several families)	Mahé, Seychelles	-4.6667	55.4333	34	66.15	0.62	0.50
77	Kaiser-Bunbury et al.[8]	(several families)	Mahé, Seychelles	-4.6667	55.4333	35	50.49	0.45	0.40
78	Kaiser-Bunbury et al.[8]	(several families)	Mahé, Seychelles	-4.6667	55.4333	23	56.99	0.40	0.32
79	Kohler[10]	(several families)	Brazil (low elevation)	-27.2700	-49.0100	24	62.99	0.87	0.52
80	Kohler[10]	(several families)	Brazil (mid elevation)	-27.2600	-49.0200	14	63.51	0.37	0.18
81	Kohler[10]	(several families)	Brazil (high elevation)	-27.2600	-49.0200	11	54.26	0.70	0.32
82	Lara[11]	(several families)	Mexico	19.2300	-98.9700	16	31.07	0.15	0.18

83	Las Casas et al.[12]	(several families)	Brazil	-7.8700	-36.4000	36	19.71	0.13	0.09
84	Lasprilla[13]	(several families)	Colombia	0.0400	-72.2700	52	75.93	0.62	0.47
85	Ollerton et al. [14]	(several families)	South Africa	-29.6167	30.1333	65	68.66	0.46	0.41
86	Vizentin-Bugoni et al.[15]	(several families)	Brazil	-23.3417	-45.1167	64	50.91	0.47	0.41
Seed dispersal									
87	Beehler[16]	(several families)	Papua New Guinea	-7.2667	146.7000	40	67.66	0.27	0.35
88	Carlo et al.[17]	(several families)	Puerto Rico (Caguana)	18.2969	-66.7819	41	44.70	0.41	0.42
89	Carlo et al.[17]	(several families)	Puerto Rico (Cialitos)	18.2583	-66.5356	54	43.38	0.46	0.50
90	Carlo et al.[17]	(several families)	Puerto Rico (Cordillera)	18.1725	-66.5919	38	29.69	0.42	0.44
91	Carlo et al.[17]	(several families)	Puerto Rico (Fronton)	18.3083	-66.5586	36	34.17	0.32	0.40
92	Frost [18]		South Africa	-28.9500	31.7500	26	74.57	0.25	0.31
93	Galetti and Pizo [19]	Birds	Southeastern Brazil	-22.8167	-47.1000	25	34.24	0.44	0.43
94	Galetti and Pizo [19]	Birds	Southeastern Brazil	-22.8167	-47.1000	64	48.92	0.36	0.40
95	Noma [20]	(several families)	Japan	30.3333	130.5000	23	35.49	0.25	0.34
96	Poulin et al. [21]	Miconia, Psichotria, Birds	Panama (Barro Colorado)	9.1500	-79.8500	23	66.80	0.23	0.22
97	Poulin et al. [21]	Miconia, Psichotria, Birds	Panama (Soberanía)	9.1667	-79.1167	24	48.29	0.20	0.25
98	Schleuning et al. [22]	(several families)	Kenya	0.2958	34.7861	121	73.90	0.30	0.36
99	Snow and Snow [23]	Birds	Trinidad.	10.7167	-61.3000	64	34.58	0.30	0.30
Epiphyte-phorophyte									
**100**	Ceballos et al. [24]	Vascular epiphyte, serveral families trees	Argentina	-26.7620	-65.3329	44	69.53	0.11	0.18

**101**	Dejean et al. [25]	Bromeliaceae, Orchidaceae	Mexico	19.38	-87.79	20	67.77	0.24	0.22
**102**	Laube et al. [26]	(several families)	Panama (San Lorenzo)	8.3	-82.1	107	20.81	0.28	0.28
**103**	Martinez-Melendez et al. [27]	Orchidaceae, Bromeliaceae	Mexico (Cerro del Quetzal)	15.72	-92.92	25	59.89	0.10	0.13
**104**	Naranjo, unpublished data	Orchidaceae	Ecuador	-3.9881	-76.1011	146	56.98	0.21	0.17
**105**	Piazzon et al. [28]	Angiosperm epiphytes	Chile (Caulin Forest)	-41.8333	-73.6000	17	64.29	0.09	0.11
**106**	Piazzon et al. [28]	Angiosperm epiphytes	Chile (Senda Darwin)	-41.8833	-73.6667	16	74.65	0.09	0.09
**107**	Piazzon et al. [28]	Angiosperm epiphytes	Chile (Llanquihue)	-41.8500	-73.5667	22	74.54	0.06	0.08
**108**	Piazzon et al. [28]	Angiosperm epiphytes	Chila (Quilar)	-41.92	-73.60	19	68.95	0.12	0.12
**109**	Sayago et al. [29]	Bromeliaceae	Mexico (Jalisco)	20.66	-103.35	62	62.89 [a]	0.23 [a]	*na*
**110**	Vergara-Torres et al. [30]	Bromeliaceae, Orchidaceae	Mexico_2	99.1139	-18.9562	16	76.67	0.1	0.09
**111**	Zhao et al. [31]	(several families)	China (Xishuangbanna)	22.01	100.8	180	16.4 [a]	0.5 [a]	*na*
Ant-myrmecophyte									
**112**	Blüthgen et al. [32]	Bromeliaceae	Venezuela	3.1333	-65.6333	17	51.39	0.78	0.54
**113**	Cabrera and Jaffe [33]	Melastomataceae	Venezuela	1.8000	-64.2333	21	37.38	0.82	0.73
**114**	Davidson et al. [34]	(several families)	Southeastern Peru	-11.8667	-71.3667	26	4.28	0.93	0.81
**115**	Dejean et al. [25]	Bromeliaceae, Orchidaceae	Mexico	*na*	*na*	60	39.46	0.23	0.34
**116**	Fiala et al. [35]	Macaranga	Borneo	*na*	*na*	16	19.30	0.66	0.47
**117**	Fiala et al. [35]	Macaranga	Borneo	*na*	*na*	18	17.81	0.64	0.45
**118**	Fiala et al. [35]	Macaranga	Borneo	*na*	*na*	12	25.00	0.54	0.53

**119**	Fiala et al. [35]	Macaranga	Sumatra	*na*	*na*	11	43.65	0.79	0.64
**120**	Fiala et al. [35]	Macaranga	West Malaysia	*na*	*na*	9	25.00	0.86	0.69
**121**	Fonseca and Ganade [36]	Ants, Myrmecophytes	Brazil	-2.4000	-59.7167	41	12.80	0.85	0.65
**122**	Yu and Davidson [37]	Cecropia	Peru	-12.8333	-69.3667	11	60.80	1.00	0.60

References

[1] Abreu, C. R. & Vieira, M. F. 2004 Os beija-flores e seus recursos florais em um fragmento florestal de Viçosa, sudeste brasileiro*. Lundiana.* **5**, 129-134.

[2] Bezerra, E. L., Machado, I. C. & Mello, M. A. 2009 Pollination networks of oil-flowers: a tiny world within the smallest of all worlds*. J. Anim. Ecol.* **78**, 1096-1101.

[3] Canela, M. B. F. 2006 Interações entre plantas e beija-flores numa comunidade de floresta atlântica montana em Itatiaia, RJ*. Tese de doutorado.* **Universidade Estadual de Campinas, Campinas**, 1-75.

[4] del Coro Arizmendi, M. & Ornelas, J. F. 1990 Hummingbirds and their floral resources in a tropical dry forest in Mexico*. Biotropica.*, 172-180.

[5] Gutiérrez, A. & Rojas-Nossa, S. V. 2001 Dinámica anual de la interacción colibrí-flor en ecosistemas altoandinos del volcán Galeras, sur de Colombia*. Tesis de grado.* **Universidad Nacional de Colombia, Bogotá**, 1-141.

[6] Ingversen, T. T. 2006 Plant–pollinator interactions on Jamaica and Dominica: The centrality, asymmetry and modularity of networks. *Msc thesis.* **University of Aarthus, Aarthus**.

[7] Kaiser-Bunbury, C. N., Muff, S., Memmott, J., Müller, C. B. & Caflisch, A. 2010 The robustness of pollination networks to the loss of species and interactions: a quantitative approach incorporating pollinator behaviour*. Ecol. Lett.* **13**, 442-452.

[8] Kaiser-Bunbury, C. N., Vázquez, D. P., Stang, M. & Ghazoul, J. 2014 Determinants of the microstructure of plant–pollinator networks. *Ecology.* **95**, 3314-3324.

[10] Kohler, G. U. 2011 Redes de interação Planta Beija-Flor em um Gradiente Altitudinal de Floresta Atlântica no Sul do Brasil.

[11] Lara, C. 2006 Temporal dynamics of flower use by hummingbirds in a highland temperate forest in Mexico. *Ecoscience.* **13**, 23-29.

[12] Las-Casas, F., Azevedo Júnior, S. & Dias Filho, M. 2012 The community of hummingbirds (Aves: Trochilidae) and the assemblage of flowers in a Caatinga vegetation. *Brazilian Journal of Biology.* **72**, 51-58.

[13] Lasprilla, L. 2003 Interações planta/beija-flor em tres comunidades vegetais da parte sul do Parque Nacional Natural Chiribiquete, Amazonas (Colombia). *Tese de doutorado.* **Universidade Estadual de Campinas, Campinas**, 1-138.

[14] Ollerton, J., Johnson, S. D., Cranmer, L. & Kellie, S. 2003 The pollination ecology of an assemblage of grassland asclepiads in South Africa. *Annals of Botany.* **92**, 807-834.

[15] Vizentin-Bugoni, J., Maruyama, P. K., Debastiani, V. J., Duarte, L. d. S., Dalsgaard, B. & Sazima, M. 2016 Influences of sampling effort on detected patterns and structuring processes of a Neotropical plant–hummingbird network. *J. Anim. Ecol.* **85**, 262-272.

[16] Beehler, B. 1983 Frugivory and polygamy in birds of paradise. *Auk.*, 1-12.

[17] Carlo, T. A., Collazo, J. A. & Groom, M. J. 2003 Avian fruit preferences across a Puerto Rican forested landscape: pattern consistency and implications for seed removal. *Oecologia.* **134**, 119-131.

[18] Frost, P. 1980 Fruit-frugivore interactions in a South African coastal dune forest. *Acta XVII Congresus Internationalis Ornithologici, Deutsches Ornithologische Gessenshaft, Berlin.* **2**, 1179-1184.

[19] Galetti, M. & Pizo, M. A. 1996 Fruit eating birds in a forest fragment in southcastern Brazil. *Ararajuba.* **4**, 71-79.

[20] Noma, N. 1997 Annual fluctuations of sapfruits production and synchronization within and inter species in a warm temperate forest on Yakushima Island. *Tropics.* **6**, 441-449.

[21] Poulin, B., Wright, S. J., Lefebvre, G. & Calderón, O. 1999 Interspecific synchrony and asynchrony in the fruiting phenologies of congeneric bird-dispersed plants in Panama. *J. Trop. Ecol.* **15**, 213-227.

[22] Schleuning, M., Blüthgen, N., Flörchinger, M., Braun, J., Schaefer, H. M. & Böhning-Gaese, K. 2011 Specialization and interaction strength in a tropical plant–frugivore network differ among forest strata. *Ecology.* **92**, 26-36.

[23] Snow, B. K. & Snow, D. 1971 The feeding ecology of tanagers and honeycreepers in Trinidad. *Auk.* **88**, 291-322.

[24] Ceballos, S. J., Chacoff, N. P. & Malizia, A. 2016 Interaction network of vascular epiphytes and trees in a subtropical forest. *Acta Oecol.* **77**, 152-159.

[25] Dejean, A., Olmsted, I. & Snelling, R. R. 1995 Tree-epiphyte-ant relationships in the low inundated forest of Sian Ka'an Biosphere Reserve, Quintana Roo, Mexico. *Biotropica.* **27**, 57-70.

[26] Laube, S. & Zotz, G. 2006 Neither Host-specific nor Random: Vascular Epiphytes on Three Tree Species in a Panamanian Lowland Forest. *Annals of Botany.* **97**, 1103.

[27] Martínez-Meléndez, N., Pérez-Farrera, M. A. & Flores-Palacios, A. 2008 Estratificación vertical y preferencia de hospedero de las epífitas vasculares de un bosque nublado de Chiapas, México. *Revista de Biología Tropical.* **56**, 2069-2086.

[28] Piazzon, M., Larrinaga, A. R. & Santamaría, L. 2011 Are Nested Networks More Robust to Disturbance? A Test Using Epiphyte-Tree, Comensalistic Networks. *PLOS ONE.* **6**, e19637.

[29] Sáyago, R., Lopezaraiza-Mikel, M., Quesada, M., Álvarez-Añorve, M. Y., Cascante-Marín, A. & Bastida, J. M. 2013 Evaluating factors that predict the structure of a commensalistic epiphyte-phorophyte network. *Proc. Biol. Sci.* **280**, 20122821.

[30] Vergara-Torres, C. A., Pacheco-Álvarez, M. C. & Flores-Palacios, A. 2010 Host preference and host limitation of vascular epiphytes in a tropical dry forest of central Mexico. *J. Trop. Ecol.* **26**, 563-570. (DOI 10.1017/S0266467410000349).

[31] Zhao, M., Geekiyanage, N., Xu, J., Khin, M. M., Nurdiana, D. R., Paudel, E. & Harrison, R. D. 2015 Structure of the epip hyte community in a tropical montane forest in SW China. *PloS one.* **10**, e0122210.

[32] Blüthgen, N., Verhaagh, M., Goitía, W. & Blüthgen, N. 2000 Ant nests in tank bromeliads—an example of non-specific interaction. *Insectes Soc.* **47**, 313-316.

[33] Cabrera, M. & Jaffe, K. 1994 A trophic mutualism between the myrmecophytic Melastomataceae *Toccoa guianensis* Aublet and an *Azteca* ant species. *Ecotropicos.* **7**, 1-10.

[34] Davidson, D. W., Snelling, R. R. & Longino, J. T. 1989 Competition among ants for myrmecophytes and the significance of plant trichomes. *Biotropica.*, 64-73.

[35] Fiala, B., Jakob, A., Maschwitz, U. & Linsenmair, K. E. 1999 Diversity, evolutionary specialization and geographic distribution of a mutualistic ant-plant complex: *Macaranga* and *Crematogaster* in South East Asia. *Biol. J. Linn. Soc.* **66**, 305-331.

[36] Fonseca, C. R. & Ganade, G. 1996 Asymmetries, compartments and null interactions in an Amazonian ant-plant community. *J. Anim. Ecol.*, 339-347.

[37] Yu, D. W. & Davidson, D. W. 1997 Experimental studies of species-specificity in cecropia–ant relationships. *Ecol. Monogr.* **67**, 273-294.

Table S2. LMM performed to contrast metrics among type of interactions. Analyses of variance to test the null hypothesis that the response means are identical across the predictor variables is included. R^2_m and R^2_c denote marginal and conditional R^2 for linear mixed model, respectively.

	Solution for effects			***Deviance change***		
Effect	***Estimate***	***SE***	***SD***	$\chi 2$	***df***	***Pr (>*** $\chi 2$***)***
**1. *Nestedness, NODF.*** $R^2_m = 0.29$, $R^2_c = 0.71$						
Fixed Effects						
Intercept	4.36	0.20		-	-	
Type of interaction	-	-	-	19.14	3	< 0.001
Seed dispersal	-0.24	0.21	-	-	-	-
Pollination	-0.35	0.20	-	-	-	-
Ant-myrmecophyte	-1.01	0.24				
Network size	-0.007	0.001	-	12.14	1	< 0.001
Random effects (Locality)						
Intercep	-	-	0.4	-	-	-
Residual	-	-	0.29	-	-	-
**2. *Network specialization (H'2).*** $R^2_m = 0.41$, $R^2_c = 0.62$						
Fixed Effects						
Intercept	0.16	0.06		-	-	
Type of interaction	-	-	-	70.70	3	< 0.0001
Seed dispersal	0.15	0.06	-	-	-	-
Pollination	0.30	0.06	-	-	-	-
Ant-myrmecophyte	0.58	0.07				
Network size	00003	0.0006	-	0.25	1	0.61
Random effects (Locality)						
Intercept	-	-	0.09	-	-	-
Residual	-	-	0.13	-	-	-
**3. *Modularity (Q).*** $R^2_m = 0.41$, $R^2_c = 0.61$						
Fixed Effects						
Intercept	0.11	0.04		-	-	
Type of interaction	-	-	-	75.78	3	< 0.01
Seed dispersal	0.21	0.05	-	-	-	-
Pollination	0.25	0.04	-	-	-	-
Ant-myrmecophyte	0.47	0.05				
Network size	0.0008	0.0005	-	2.22	1	0.14
Random effects (Locality)						
Intercept	-	-	0.06	-	-	-
Residual	-	-	0.09	-	-	-

β, parameter value; *SE,* standard error*;* *SD*, standard deviation. Missing levels of type of interaction (epiphyte–phorophyte) is included in the intercept.

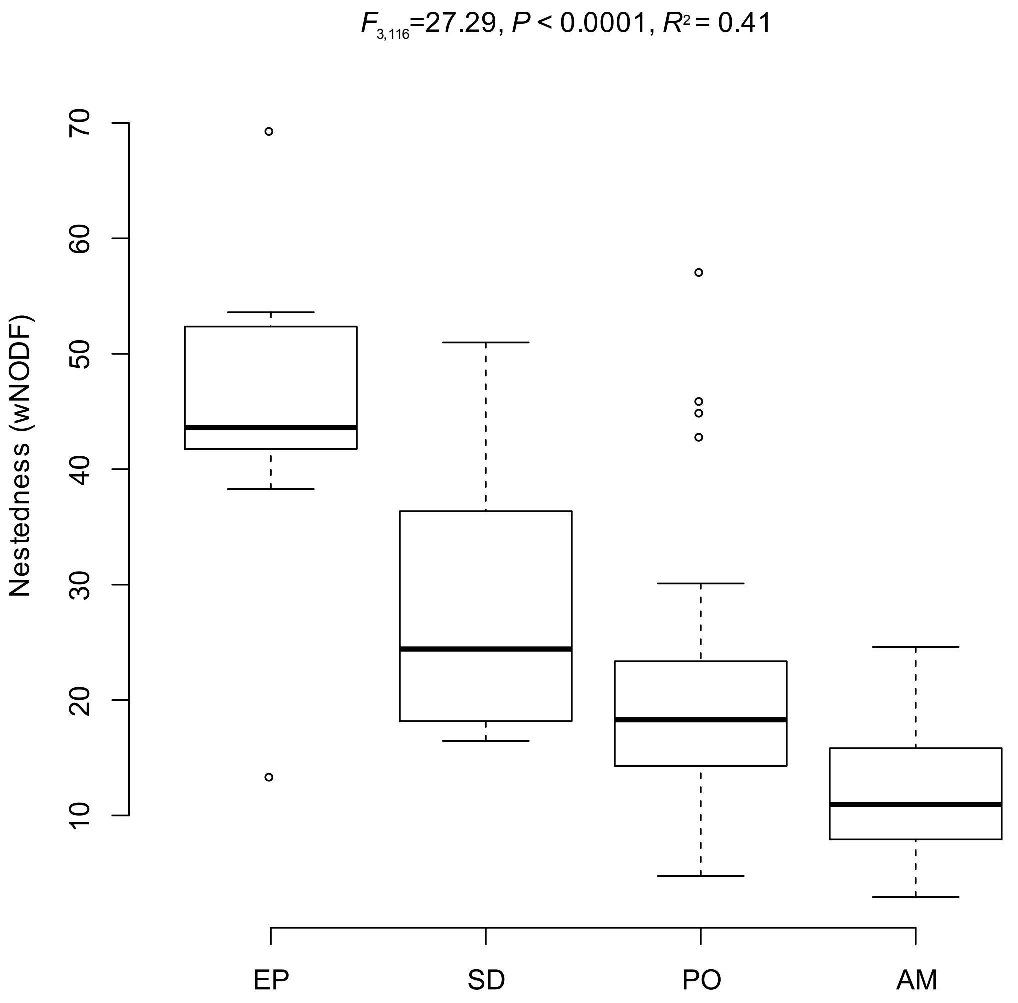

Figure S1. Distribution of weighted NODF values across network interaction types: commensalistic epiphyte–phorophyte (EP), seed dispersal (SD), pollination (PO) and ant-myrmecophyte (AM) networks. Linear models result for testing the effects of type of interaction is displayed above each graph.
